# Supplementary material for: Akaganeite nanorices deposited muscovite mica surfaces as sunlight active green photocatalyst
Source: R Soc Open Sci. 2019 Mar 27;6(3):182212. doi: 10.1098/rsos.182212 (PMC6458388; doi:10.1098/rsos.182212)
Supplement: XRF Elementary Analysis [file rsos182212supp1.docx]

**Supplementary Data**

XRF elementary analysis is given below

Fig. S1. XRF data of (a) akaganeite NPs (b) Mica (c) ANPM


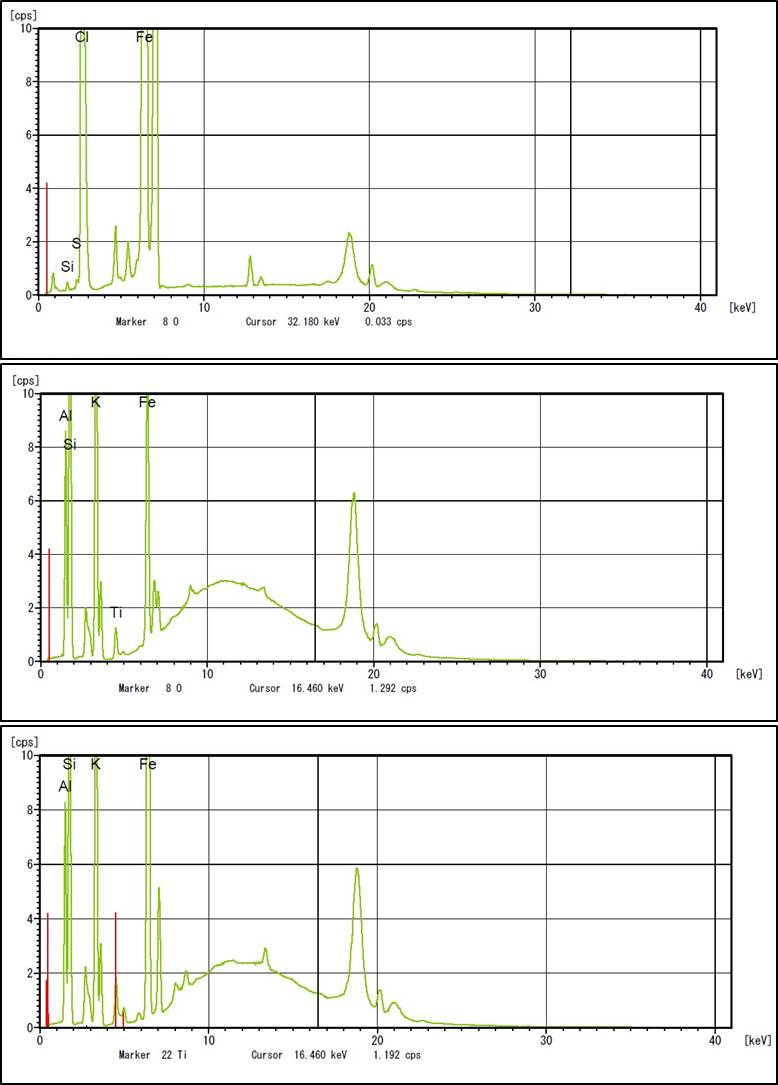


(a)

(b)

(c)
